# A systematic review protocol for assessing equity in clinical practice guidelines for traumatic brain injury and homelessness

**DOI:** 10.3389/fmed.2022.815660

**Published:** 2022-07-22

**Authors:** Vincy Chan, Maria Jennifer Estrella, Jessica Babineau, Angela Colantonio

**Affiliations:** ^1^KITE-Toronto Rehabilitation Institute, University Health Network, Toronto, ON, Canada; ^2^Institute of Health Policy, Management and Evaluation, University of Toronto, Toronto, ON, Canada; ^3^Rehabilitation Sciences Institute, University of Toronto, Toronto, ON, Canada; ^4^Department of Occupational Science and Occupational Therapy, University of Toronto, Toronto, ON, Canada; ^5^Library and Information Services, University Health Network, Toronto, ON, Canada; ^6^The Institute for Education Research, University Health Network, Toronto, ON, Canada

**Keywords:** clinical practice guideline, traumatic brain injury, concussion, homeless, health inequity, evidence-based practice, knowledge synthesis, disadvantaged populations

## Abstract

**Background:**

When used optimally, clinical practice guidelines (CPGs) can reduce inappropriate variations in practice, improve application of research to practice, and enhance the quality of healthcare. However, a common criticism, despite its potential, is the lack of consideration for equity and disadvantaged populations.

**Objectives:**

This protocol is for a systematic review of CPGs for traumatic brain injury (TBI) and homelessness that aims to assess (1) the extent to which evidence regarding TBI and homelessness is integrated in CPGs for homelessness and TBI, respectively, and (2) equity considerations in CPGs for TBI and homelessness.

**Methods and analysis:**

The methodology for this review is guided by the PRISMA-P, validated search filters for CPGs, and methodological guides to searching systematic reviews and gray literature. CPGs will be identified from (a) databases for peer-reviewed literature (MEDLINE, Embase, CINAHL, and PsycInfo), (b) targeted websites and Google Search for gray literature, and (c) reference lists of peer-reviewed and gray literature that meet the eligibility criteria. Searching for gray literature, including from guideline-specific resources, is a critical component of this review and is considered an efficient approach to identifying CPGs, given the low precision of searching peer-reviewed databases. Two independent reviewers will screen all articles based on pre-determined eligibility criteria. A narrative synthesis will be conducted to identify the proportion of CPGs that integrate evidence about TBI and homelessness and how TBI and homelessness is or is not integrated in CPGs. Quality appraisal will take the form of an equity assessment of CPGs and will be completed independently by two reviewers.

**Conclusion:**

This protocol outlines the methodology for a systematic review of CPGs for TBI and homelessness. The resulting systematic review from this protocol will form an evidence-based foundation to advance CPGs for individuals with lived experience of TBI and homelessness.

**Systematic review registration:**

identifier: CRD42021287696.

## Introduction

Traumatic brain injury (TBI) research has gained momentum in recent years, owing to the condition being a growing public health concern (1–3). Defined as “an alteration in brain function or other evidence of brain pathology caused by an external force,” (4) TBI remains a leading cause of death and disability among all trauma-related injuries, across all ages and in all countries (5, 6). The consequences of TBI, regardless of severity, are long-lasting, leaving survivors of TBI with significant physical, emotional, and cognitive disabilities (7, 8) and costing the international economy an estimated US$400 billion annually (9). The immense global challenge caused by TBI has led to research efforts to increase understanding about TBI, reduce its burden, and improve the quality of life of, and healthcare for, survivors of TBI (9).

The development and dissemination of clinical practice guidelines (CPGs) is a known method of translating research findings to practice, including in the field of TBI (10–12). CPGs are “statements that include recommendations intended to optimize patient care that are informed by a systematic review of evidence and an assessment of the benefits and harms of alternative care options” (13). When used optimally, CPGs can reduce inappropriate variations in practice, improve application of research to practice, and enhance quality and safety of healthcare (13); however, CPGs are often criticized for focusing on effectiveness and cost-effectiveness of treatment (14). Another criticism is the lack of consideration for equity (14–16) [i.e., the fair distribution and access to resources and opportunities (17)] and health inequities or “differences in health that are not only unnecessary and avoidable, but in addition, are considered unfair and unjust” (18). In effect, CPG interventions and recommendations may not always be available, cost-effective, or beneficial for all population groups, specifically disadvantaged groups who experience substantial health inequities (15). We acknowledge the possible stigma that accompanies the term *disadvantaged*, marginalized, or underserved (19). This paper will use the term “*disadvantaged group*” to capture the lack of fair opportunities that put these individuals in a disadvantaged position (18) and to remain consistent with the language used by the GRADE Working Group (14, 19).

One such disadvantaged group is individuals with lived experience of homelessness (20, 21), where TBI has been found to be disproportionately prevalent (22). Individuals experiencing homelessness suffer significant health inequities that are exacerbated by social determinants of health (e.g., poverty, adverse experiences and trauma, lack of education, unemployment, domestic violence, and social disconnection) (20, 21). Such health inequities include poorer mental and physical health, higher morbidity and mortality, greater use of acute hospital services, and reduced likelihood of accessing primary and preventive health services (21). The latter subsequently leads to delayed diagnoses, poor control of health conditions, and hospitalization for preventable conditions (21). These health inequities speak to the need to view homelessness as both a medical and a social issue (21), and for CPGs to integrate evidence regarding homelessness into recommendations to not only manage adverse outcomes associated with TBI but also take into account the differences in injury experience and needs of individuals experiencing homelessness. Failure to integrate research findings that may illuminate the inequities experienced by this disadvantaged group could lead to lack of prioritization for their needs and misallocation of resources, overestimation or underestimation of treatment effectiveness, lack of consideration for specific outcomes that are perceived valuable, and continued barriers to accessing care (14).

In recognizing the role of guidelines in promoting health equity, the GRADE Working Group published the GRADE Equity Guidelines Series to guide efforts to incorporate considerations regarding health equity in developing and evaluating CPGs (19, 23–25). Unfortunately, most studies included in CPGs for TBI are population-based and fail to consider the diversity in patient characteristics, thereby promoting a one-size-fits-all approach to care (9).

This protocol outlines the methodology for a systematic review of CPGs for TBI and homelessness. Through conducting a preliminary search, we found that clinical guidelines for homelessness have also been developed to provide recommendations for all individuals experiencing homelessness. As such, the primary objective is to assess the extent to which evidence about homelessness is integrated in CPGs for TBI and the extent to which evidence about TBI is integrated in CPGs for homelessness. The secondary objective of the systematic review is to assess equity considerations in CPGs for TBI and homelessness. Findings from the systematic review will provide an evidence-based foundation to advance CPGs for individuals with lived experience of TBI and homelessness.

## Methods and analysis

This protocol is guided by the Preferred Reporting Items for Systematic Review and Meta-Analysis (PRISMA) Protocols (PRISMA-P) (26) and a methodological guide to systematic review of CPGs (27). The reporting of the systematic review search strategy will follow the PRISMA extension for searching (PRISMA-S) (28) and the reporting of the systematic review will follow the PRISMA Equity Extension (29). This protocol is registered on PROSPERO (CRD42021287696).

### Search strategy

We will search the following for CPGs: (a) databases for peer-reviewed literature, (b) targeted websites and Google Search for gray literature, and (c) reference lists of peer-reviewed and gray literature that meet the inclusion criteria.

#### Peer-reviewed literature

The development of the search strategy for databases was informed by (a) a validated search for retrieving CPGs from MD Anderson Cancer Centre Library (MDACCL) (30) and (b) search strategies of scoping or systematic reviews of CPGs, TBI, and/or homelessness (11, 12, 31–33). Three concepts, (A) CPG, (B) TBI, and (C) homelessness, were developed to form the final search structure, (A+B) OR (A+C), that will be used to search each database. No date or language limits will be placed on the search strategies, however, where possible, we will exclude animal studies and conference abstracts. Supplementary Material 1 presents the search strategy that was developed for the MEDLINE® ALL (in Ovid, including Epub Ahead of Print, In-Process & Other Non-Indexed Citations, Ovid MEDLINE® Daily) database, which will be translated to Embase and Embase Classic (Ovid), CINAHL (EBSCO), and APA PsycInfo (Ovid). This search strategy was developed with an Information Specialist (JB) and team members with research and subject-matter expertise relevant to TBI and homelessness (VC, MJE, AC).

#### Gray literature

The search strategy for gray literature was informed by Goldin et al.'s methodology on applying systematic review search methods to gray literature (34). Gray literature for this systematic review is defined as CPGs that are identified outside of the peer-reviewed literature. They will be identified by searching targeted websites and Google Search.

Targeted websites include those from guideline development organizations and CPG databases/repositories, health technology assessment agencies, medical or allied health professional associations, and brain injury and housing organizations. *Gray Matters: A Practical Tool for Searching Health-Related Gray Literature* developed by the Canadian Agency for Drugs and Technologies in Health (CADTH; hereafter referred to as “*Gray Matters*”) (35) will be used to identify targeted websites. Additional websites not captured by *Gray Matters* will be identified by the research team (VC, MJE, JB, AC) and through consultation with stakeholders of the systematic review, which include front-line staff and service providers in the housing and brain injury sectors; health professionals who provide care for individuals with TBI and/or lived experience of homelessness; and researchers who conduct research and develop guidelines on TBI and homelessness. Google Search will also be used to identify additional relevant websites and gray literature.

The targeted websites will be searched by entering keywords for concept (A) CPGs, (B) TBI, and (C) homelessness in the search bar. Websites without a search bar will be manually reviewed for relevant gray literature. Supplementary Material 2 presents the keywords that will be used to develop the search strategies for each targeted website and Google Search, applying the search functionalities of the websites. The search structure for peer-reviewed literature will also be applied to the gray literature search: (A+B) OR (A+C). Supplementary Material 3 presents targeted websites identified by the research team that are not captured by Gray Matters and will be expanded upon consultation with stakeholders.

#### Reference list

Reference lists of scoping and systematic reviews and CPGs identified from the databases and the gray literature search will be manually reviewed.

### Study selection

#### Eligibility criteria

Only CPGs for TBI or CPGs for homelessness will be included and they must meet the eligibility criteria outlined in the PICAR Statement (27) presented in Table 1. The PICAR Statement for systematic review of CPGs is adapted from the PICO statement for systematic review of interventions (27).

**Table 1:** PICAR statement for eligibility criteria.

	**Clinical practice guidelines for traumatic brain injury**	**Clinical practice guidelines for homelessness**
**Inclusion criteria**		
P: Population, clinical indicator(s), and conditions(s)	– TBI of any cause and injury severity	– Individuals experiencing homelessness – Individuals with lived experience of homelessness – Individuals at risk of homelessness
I: Intervention(s)	Any intervention	
C: Comparator(s), Comparison(s), and (Key) Content	Any comparator or comparison, no ‘key' CPG content is of interest (i.e., all content will be considered)	
A: Attributes of CPG	– CPGs are explicitly evidence-based (i.e., CPG must show evidence a literature search was performed) – Only the latest version of the CPG will be considered – The complete CPG must be available No restrictions will be made based on other attributes such as language, year of publication, country of publication, age (e.g., pediatrics, adults), population (e.g., veterans, athletes), setting (e.g., rehabilitation, acute care, shelters), intended end-user	
R: Recommendation Characteristics and “Other” Considerations	– At least one evidence-based recommendation must be included – CPG must use a system to rate the level of evidence behind the recommendation and the appraisal tool must be specified	
**Exclusion criteria**		
P: Population, clinical indicator(s), and conditions(s)	– CPGs focused on the broader brain-injured population (e.g., acquired brain injury) or individuals with cognitive impairment without specific recommendation(s) for TBI	– CPGs not explicitly stated as being for homelessness, individuals experiencing homelessness, individuals with lived experience of homelessness, or individuals at risk of homelessness
I: Intervention(s)	N/A	
C: Comparator(s), Comparison(s), and (Key) Content	N/A	
A: Attributes of CPG	– Summaries of guidelines, editorials – Adaptations of existing guidelines for audiences other than intended end-user (e.g., guidelines for practitioners adapted for patients), translation of guidelines	
R: Recommendation Characteristics and “Other” Considerations	– Recommendations are not rated and are not based on evidence from the literature	

*CPG, Clinical practice guideline; N/A, Not applicable; TBI, Traumatic brain injury*.

#### Peer-reviewed literature

EndNote X8 (36) will be used for reference management and Covidence (37) will be used for de-duplication and study selection. Two independent reviewers will screen all articles retrieved from the search strategy based on the above eligibility criteria. At the title and abstract screen, articles that focus on (a) the broader brain-injured population without specific mention of TBI or (b) housing without specific mention of homelessness will also be considered for full-text review to confirm that the guidelines focus on the TBI population or on homelessness. Scoping and systematic reviews of CPGs for TBI or homelessness will also be included in the title and abstract screen and their reference lists will be manually searched to identify additional CPGs not retrieved using the above search strategy. Any additional guidelines retrieved will be subject to the above inclusion and exclusion criteria.

Prior to formal title and abstract screening, a pilot screen of 20 articles will be conducted until a minimum of 80% agreement using the kappa statistic is achieved between the two reviewers. Similarly, prior to formal full-text screening, a pilot of 10% of full-text articles will be conducted until a minimum 80% agreement is achieved between the two reviewers. Non-English language abstracts will be assessed using the English full-text translation, DeepL Translate, Google Translate, or reviewers with knowledge of the language. Any discrepancies will be resolved by consensus or consultation with a third reviewer. The PRISMA flow chart (38) will be completed to illustrate the study selection process, including the number of English and non-English articles retrieved and included in the review.

#### Gray literature

Two independent reviewers will review the Gray Matters checklist and the first ten pages of Google Search results to identify potentially relevant websites using the title and/or short text underneath the title. The reviewers will then document the name and link to each website/organization and the date the website/organization was identified in an Excel file to develop a list of unique targeted websites for searching. These websites will be searched by two independent reviewers for potentially relevant CPGs using the search strategy outlined in Supplementary Material 2. The date on which each website was searched and the authors, titles, and links of the potentially relevant CPGs will be documented in the Excel file to generate a list of unique articles to review. This list will be compared to CPGs identified through the search for peer-reviewed literature and duplicates will be removed prior to screening.

Two independent reviewers will screen all articles retrieved from the targeted websites based on the eligibility criteria outlined in Table 1. At the title and abstract screen, the executive summaries and/or table of contents will be reviewed if an abstract is not available. Similar to the study selection of peer-reviewed literature, CPGs that focus on (a) the broader brain-injured population without specific mention of TBI or (b) housing without specific mention of homelessness will also be considered for full-text review to confirm that the guidelines focus on the TBI population or on homelessness. All decisions will be documented in an Excel file and a numeric code will be assigned to each article: 1 = include or maybe (i.e., it is unclear whether it meets eligibility criteria) and 0 = exclude. The screening of full-text articles will also be documented in an Excel file using the numeric code of 1 = include and 0 = exclude. The reason for excluding any full-text articles will also be documented in the Excel file. Supplementary Material 4 presents the Excel file that will be used to document the study selection process for gray literature; this file may be adapted during the systematic review.

As with the study selection process for peer-reviewed literature, a pilot screen of 20 CPGs will be conducted at the title and abstract screen and 10% of full-texts will be conducted until a minimum 80% agreement is achieved between the two reviewers. Any discrepancies will be resolved by consensus or consultation with a third reviewer. Similar to the selection process for peer-reviewed literature, non-English language reports will be assessed using any available English translations, DeepL Translate, Google Translate, or reviewers with knowledge of the language. The study selection process for gray literature will be added to the PRISMA flow chart that will be generated for peer-reviewed literature (38), including the number of English and non-English reports identified and included in the review. References will be added to EndNote X8 (36) for reference management.

### Data extraction and synthesis

The data extraction and synthesis plan presented in this protocol was adapted from Tannenbaum et al. (39), who examined sex and gender considerations in Canadian CPGs. First, the text and reference lists of the CPGs will be searched for (a) keywords describing TBI and homelessness that are consistent with the search strategy for peer-reviewed literature and gray literature and (b) content consistent with the definitions of TBI and homelessness, both displayed in Table 2. This will enable us to categorize the guidelines into (a) text-positive or (b) text-negative.

**Table 2:** Keywords and definitions for traumatic brain injury and homelessness.

	**Keywords describing TBI/homelessness or content consistent with the definition of TBI/homelessness**
Traumatic brain injury	Keywords: brain injury or concussion or brain trauma or head injury or head trauma Definition (4): An alteration in brain function, or other evidence of brain pathology, caused by an external force
Homelessness	Keywords: homelessness or roofless or marginally housed or precariously housed or unstably housed or provisionally accommodated or houseless or shelters Definitions of typology of homelessness that encompasses the following physical living situations (40): a) Unsheltered: Individuals who lack housing and are not accessing shelters – Public or private spaces without consent or contract – Places not intended for permanent human habitation b) Emergency sheltered: Individuals who cannot secure permanent housing and are accessing shelters or other system supports – Emergency overnight shelters for people who are homeless – Shelters for individuals/families impacted by family violence – Emergency shelter for people fleeing a natural disaster or destruction of accommodations due to fires, floods, etc. c) Provisionally accommodated: Individuals without permanent shelter and are accessing accommodations that offer no prospect of permanence – Interim housing – Living temporarily with others – Accessing short-term, temporary rental without security of tenure – Living in institutional care and lack housing arrangements – Accommodation/reception centers for recently arrived immigrant and refugees d) At risk of homelessness: Individuals or families whose current housing situations are dangerously lacking security or stability – People at imminent risk of homelessness – Individuals and families who are precariously housed

*TBI, Traumatic brain injury*.

Text-positive *CPGs for TBI* are those that contain at least one of the keywords for or content consistent with homelessness in the text (i.e., body) of the guidelines. Similarly, text-positive *CPGs for homelessness* are those that contain at least one of the keywords for or content consistent with TBI in text of the guidelines. Text-negative *CPGs for TBI* are those that do not include any of the keywords for or content consistent with homelessness in the body of the guidelines, while text-negative *CPGs for homelessness* are those that do not include any of the keywords for or content consistent with TBI in the body of the guidelines.

Text-positive and text-negative guidelines will be synthesized separately. Text-positive *TBI CPGs* will be categorized into one of the following categories: (1) guideline specifically recommends evidence-based diagnostic, management, or treatment approaches for individuals experiencing homelessness or with lived experience of homelessness; (2) guideline acknowledges or makes reference to data (e.g., epidemiologic, risk factors, outcome) regarding individuals experiencing homelessness or with lived experience of homelessness only, without recommendations; or (3) guideline mentions individuals experiencing homelessness or with lived experience of homelessness without context related to the literature or recommendations. Text-positive *CPGs for homelessness* will be categorized into one of the following categories: (1) guideline specifically recommends evidence-based diagnostic, management, or treatment approaches for individuals with TBI; (2) guideline acknowledges or makes reference to data (e.g., epidemiologic, risk factors, outcome) regarding individuals with TBI only, without recommendations; or (3) guideline mentions individuals with TBI without context related to the literature or recommendations. Data (i.e., quotes) that are used to categorize the guidelines will be extracted.

Text-negative *TBI CPGs* will be categorized into one of two categories: (1) reference lists contain articles that include keywords for homelessness but the text of the guideline does not contain any keywords for or content consistent with the definition of homelessness or (2) no article in the reference list includes keywords for homelessness. Text-negative *CPGs for homelessness* will be categorized into one of two categories: (1) reference lists contain articles that include keywords for TBI but the text of the guideline did not contain any keywords for or content consistent with the definition of TBI or (2) no article in the reference list includes keywords for TBI. Data (i.e., the reference) that are used to categorize the guideline into category one will be extracted.

Two independent reviewers will complete the data extraction and synthesis. Similar to the study selection process, a pilot extraction and synthesis of 10% of guidelines will be conducted until a minimum 80% agreement using the kappa statistic is achieved between the two reviewers. Discrepancies will be resolved through consensus or consultation with a third reviewer.

### Analysis

A narrative synthesis will be conducted, informed by tools and techniques in the Guidance on the Conduct of Narrative Synthesis in Systematic Reviews (41):

1. Groupings and clusters: In addition to categorizing the text-positive and text-negative guidelines into the groups above, the guidelines will also be grouped by other characteristics, with the number and types of groupings to be refined during data synthesis. Some groupings that will be explored include (a) the type of guideline—TBI vs. homelessness, (b) country the guideline was developed, (c) year of publication, (d) focus of the guideline—e.g., early management, rehabilitation, etc., (e) population the guideline was developed for—e.g., pediatrics vs. adults, (f) target audience (e.g., clinicians, allied health professionals, schools).
2. Tabulation: The number of guidelines in each of the groups will be identified, with the primary outcomes of interest being the proportion of text-positive guidelines for TBI (i.e., the number guidelines for TBI that were text-positive divided by the total number of guidelines for TBI included in the review) and homelessness (i.e., the number guidelines for homelessness that were text-positive divided by the total number of guidelines for homelessness included in the review).
3. Qualitative analysis: Content analysis of text-positive and text-negative guidelines (42) will be conducted to address a secondary outcome of this review, which is to understand how homelessness is or is not integrated in CPGs for TBI, and how TBI is or is not integrated in CPGs for homelessness. This analytical method is appropriate, as it allows both quantification and categorization of data (41).

### Quality appraisal

Quality appraisal will take the form of an equity assessment of CPGs. The equity lens from Dans et al. (14) and the equity extension from the GRADE working group will be used to assess equity in the CPGs identified in the systematic review (19, 23–25). Table 3 presents the five criteria proposed by Dans et al. to evaluate how well CPGs address equity (14) and the considerations for equity in the GRADE methodology for developing clinical, public health, and health system guidelines (hereafter referred to as the “GRADE Equity Guideline”) from the GRADE working group (23). Any technical, methodological, or supporting documents associated with the CPGs included in the review will be retrieved to better inform the quality appraisal process (27). Quality appraisal will be completed by two independent reviewers, with a pilot assessment of 10% of guidelines until a minimum 80% agreement using the kappa statistic is achieved between the two reviewers. Discrepancies will be resolved through consensus or consultation with a third reviewer.

**Table 3:** Quality appraisal checklist to assess equity in clinical practice guidelines.

**Evaluation tools**	**Yes/no/unclear/not reported**
**Assessing equity in clinical practice guidelines (14)**	
1. Discusses the burden of disease in disadvantaged populations.	
2. Discusses differences between disadvantaged and privileged populations, in terms of the biology of the disease, adherence, and baseline risks.	
3. Values (of the intervention/outcome) assessed in guideline development panels through consultations with disadvantaged populations, involvement of their caregivers, reference to relevant search, or transparent reflection.	
4. Discusses barriers to implementation in disadvantaged populations, and identifies strategies to overcome these barriers.	
5. Plans for monitoring disadvantaged groups according to PROGRESS-plus elements.	
**GRADE equity guideline: equity extension of the guideline development checklist (23)**	
1. Setting priorities a. Dedicates part of or entire guideline to the care of disadvantaged populations.	
2. Guideline group membership a. Includes representatives of the disadvantaged populations in the different guideline groups, particularly the voting panel b. Ensures the method for recruitment of group members considers representatives of all relevant disadvantaged populations c. Recruits a methodologist who is familiar with and mindful of equity issues d. Ensures the chair of the voting panel is familiar with equity issues.	
3. Identifying the target audience(s) a. Specifies relevant disadvantaged populations when identifying the target audience(s) b. Involves representatives of the disadvantaged populations when identifying the target audience(s).	
4. Generating the guideline questions a. Considers equity when specifying elements of the PICO questions b. Considers “good-practice statements” that could help address equity issues.	
5. Considering the importance of outcomes and interventions a. Involves representatives of disadvantaged populations in rating the importance of interventions and outcomes b. Searches selected databases for outcomes rated as important by disadvantaged populations c. Considers separate recommendations for disadvantaged populations if their values and preferences are thought to differ substantively to the point of affecting the strength and/or direction of the recommendation.	
6. Deciding what evidence to include and searching for evidence a. Seeks evidence specific to disadvantaged populations, for example, baseline risks specific to those groups b. Considers including evidence derived from fields other than health (e.g., social science) that address disadvantaged populations c. Searches literature published in the language relevant to the disadvantaged population.	
7. Summarizing the evidence and considering additional information a. Considers the PROGRESS-plus elements when synthesizing the evidence b. Follows the PRISMA-equity statement when reporting the systematic reviews c. Considers information on resource use, cost, effect on equity, feasibility, and acceptability from the perspective of disadvantaged populations.	
8. Wording of recommendations a. Specific in defining the population to maximize the understanding that it applies to a disadvantaged population (when applicable) b. Includes the necessary remarks following the recommendation to ensure its appropriate implementation in disadvantaged populations c. Ensures that language is used carefully so that the recommendation does not stigmatize already disadvantaged populations.	
9. Evaluation and use a. Produces tools to facilitate implementation and use among disadvantaged populations b. Monitors and audits implementation and use among disadvantaged populations.	

*PICO, Population, Intervention, Comparison, Outcome; PRISMA,Preferred Reporting Items for Systematic Review and Meta-Analysis; PROGRESS-plus, Place of residence, Race/ethnicity/culture/language, Occupation, Gender/sex, Religion, Education, Socioeconomic status, or Social capital, with “plus” referring to other relevant characteristics (e.g., age, disability, sexual orientation, etc.) (43)*.

Results of the quality appraisal will complement findings from the review and will be integrated in the analysis. As such, no CPG will be excluded from this review based on the quality appraisal. We believe this is an appropriate quality appraisal compared to more traditional assessments tools for CPGs, such as the Appraisal of Guidelines for Research and Evaluation (AGREE) Instrument, as the focus of the systematic review is to assess the extent to which evidence regarding homelessness is integrated in TBI CPGs and the extent to which TBI is integrated in CPGs for homelessness. It also directly addresses the secondary aim of assessing equity considerations in CPGs.

## Discussion

This protocol outlines the methodology for a systematic review of CPGs for TBI and homelessness. It is guided by the PRISMA-P (26), validated search filters for CPGs (30), and methodological guides to systematic reviews (27) and gray literature searching (34). In particular, this protocol outlines a transparent, rigorous, and systematic search strategy to identify gray literature to increase replicability. Searching for gray literature, including from guideline-specific resources, is a critical component of this review and is considered an efficient approach to identifying CPGs, given the low precision of searching databases for peer-reviewed CPGs (30).

However, we acknowledge limitations of this protocol. First, this systematic review will determine if and how CPGs for TBI and homelessness consider homelessness and TBI, respectively; it will not systematically search for and review available research evidence on TBI and homelessness. As such, a separate systematic review of evidence on homelessness and TBI is encouraged to identify missed opportunities to integrate evidence regarding homelessness into CPGs for TBI and evidence regarding TBI into CPGs for homelessness. Furthermore, we recognize the risk of publication bias, as only publicly available CPGs will be included in the review. It is plausible that organizations may develop evidence-based CPGs that are not publicly available and these will be missed, unless identified through the gray literature search. Furthermore, while we will not place any restrictions on the language, year, and country of the CPGs, we recognize that non-English language CPGs may still be missed in this review. Finally, we acknowledge that the quality appraisal tool selected for the systematic review will not assess the methodological rigor of the CPGs. However, we believe the quality appraisal selected for this study is appropriate as it directly addresses the aims of this review.

Despite these limitations, the systematic review that will be produced from this protocol is a critical first step to addressing care for individuals with lived experience of homelessness and TBI. Assessing the extent to which evidence about homelessness is integrated in CPGs for TBI and the extent to which evidence about TBI is integrated in CPGs for homelessness, along with equity considerations in CPGs for TBI and homelessness, will form an evidence-based foundation to advance CPGs for individuals with lived experience of TBI and homelessness.

## Ethics Statement

Research Ethics Board approvals are not required for the systematic review as only publicly available data will be analyzed. Findings will be disseminated in the form of a peer-reviewed publication and presentations at scientific conferences and to stakeholders of this research.

## Author contributions

VC and AC conceptualized the study. VC, MJE, and JB developed the search strategy. VC formulated the design. VC and MJE drafted the manuscript. All authors critically reviewed the manuscript and approved the final manuscript.

## Funding

This work has been supported in part by the Canada Research Chairs Program (Fund Number N/A) and the Ontario Ministry of Health and Long-Term Care, Ministry Grant #725A.

## Conflict of interest

The authors declare that the research was conducted in the absence of any commercial or financial relationships that could be construed as a potential conflict of interest.

## Author disclaimer

The views expressed in the publication are the views of the authors and do not necessarily reflect those of the Ministry of Health and Long-Term Care.

## Publisher's note

All claims expressed in this article are solely those of the authors and do not necessarily represent those of their affiliated organizations, or those of the publisher, the editors and the reviewers. Any product that may be evaluated in this article, or claim that may be made by its manufacturer, is not guaranteed or endorsed by the publisher.
