# Supplementary material for: A systematic review protocol for assessing equity in clinical practice guidelines for traumatic brain injury and homelessness
Source: Front Med (Lausanne). 2022 Jul 22;9:815660. doi: 10.3389/fmed.2022.815660 (PMC9353519; doi:10.3389/fmed.2022.815660)
Supplement: Supplementary file 1 [file Table_1.pdf]

Supplementary Material 1. Search Strategy for Medline ALL (Ovid)

1 exp Homeless persons/
2 homeless*.tw,kf.
3 Roofless*.tw,kf.
4 (Marginal* adj3 hous*).tw,kf.
5 (precarious* adj3 hous*).tw,kf.
6 (unstabl* adj3 hous*).tw,kf.
7 (instab* adj3 hous*).tw,kf.
8 (interim* adj3 hous*).tw,kf.
9 (temporary adj3 (liv* or hous*)).tw,kf.
10 ((liv* or sleep* or stay or emergenc*) adj3 shelter??).tw,kf.
11 houseless*.tw,kf.
12 unsheltered.tw,kf.
13 rough sleeper?.tw,kf.
14 rough sleeping.tw,kf.
15 provisionally accommodat*.tw,kf.
16 or/1-15
17 exp Brain Injuries/
18 exp Brain Injuries, Traumatic/
19 exp Brain Concussion/
20 Craniocerebral Trauma/
21 tbi*2.tw,kf.
22 mtbi*2.tw,kf.
23 concuss*.tw,kf.
24 postconcuss*.tw,kf.
25 ((head* or brain* or cerebr* or crani* or skull* or intracran*) adj2 (injur* or trauma* or damag* or wound* or swell* or oedema* or edema* or fracture* or contusion* or pressur*)).tw,kf,jw.
26 ((brain* or cerebr* or intracerebr* or crani* or intracran* or head* or subdural* or epidural* or extradural*) adj (haematoma* or hematoma* or hemorrhag* or haemorrhag* or bleed*)).tw,kf.
27 or/17-26
28 guidleline.pt. or exp guideline/ or exp practice guideline/ or exp Consensus/ or exp Consensus Development Conference, NIH/ or exp Consensus Development Conference/ or (consensuses or consensus or position statement or position statements or practice parameter or practice parameters or "appropriate use criteria" or appropriateness criteria or guidance statement or guidance statements or guideline or guidelines or bulletin).ti,bt.
29 (16 or 27) and 28
30 29 not (exp animals/ not exp humans/)
