# Supplementary material for: A systematic review protocol for assessing equity in clinical practice guidelines for traumatic brain injury and homelessness
Source: Front Med (Lausanne). 2022 Jul 22;9:815660. doi: 10.3389/fmed.2022.815660 (PMC9353519; doi:10.3389/fmed.2022.815660)
Supplement: Supplementary file 2 [file Table_2.pdf]

Supplementary Material 2. Keywords for Targeted Websites and Google Search Engine.

**Concept**	**Keyword**
A. Clinical Practice Guidelines	consensuses or consensus or position statement or position statements or practice parameter or practice parameters or "appropriate use criteria" or appropriateness criteria or guidance statement or guidance statements or guideline or guidelines or bulletin
B. Traumatic brain Injury	brain injury or concussion or brain trauma or head injury or head trauma or TBI
C. Homelessness	homelessness or roofless or marginally housed or precariously housed or unstably housed or provisionally accommodated or houseless or shelters

Final search structure: (A+B) OR (A+C)
