# Supplementary material for: A systematic review protocol for assessing equity in clinical practice guidelines for traumatic brain injury and homelessness
Source: Front Med (Lausanne). 2022 Jul 22;9:815660. doi: 10.3389/fmed.2022.815660 (PMC9353519; doi:10.3389/fmed.2022.815660)
Supplement: Supplementary file 3 [file Table_3.pdf]

Supplementary Material 3. Targeted Websites

#	Website Name/Organization	Link
1	Epistemonikos	https://www.epistemonikos.org/
2	National Collaborating Centre for Chronic Conditions	https://www.guidelinecentral.com/summaries/organizations/national-collaborating-centre-for-chronic-conditions/
3	MD Anderson Cancer Centre	https://mdanderson.libguides.com/c.php?g=564859&p=3891068#gsc.tab=0
4	American Academy of Physical Medicine and Rehabilitation	https://www.aapmr.org/
5	Canadian Alliance to End Homelessness	https://caeh.ca/
6	Canadian Housing First Toolkit	https://housingfirsttoolkit.ca/
7	Centre for Urban Health Solutions	https://maphealth.ca/
8	Cochrane Methods Equity Homeless Health Guidelines	https://methods.cochrane.org/equity/projects/homeless-health-guidelines
9	Evidence Exchange Network for Mental Health and Addictions	https://www.eenet.ca/
10	Mental Health Commission of Canada	https://mentalhealthcommission.ca/
11	Model Systems Knowledge Translation Center	https://msktc.org/
12	National Association of State Head Injury Administrators	https://www.nashia.org/
13	National Health Care for the Homeless Council	https://nhchc.org/
14	National Institute on Disability, Independent Living and Rehabilitation Research (NIDILRR)	https://acl.gov/about-acl/about-national-institute-disability-independent-living-and-rehabilitation-research
15	Ruff Institute of Global Homelessness	https://ighomelessness.org/
16	The Center for Brain Injury Research and Training	https://cbirt.org/
17	The Homeless Hub	https://www.homelesshub.ca/

18	Toronto Alliance to End Homelessness	https://taeh.ca/
19	Toronto Mental Health and Addictions Supporting Housing Network	https://tosupportivehousing.ca/
20	Wellesley Institute	https://www.wellesleyinstitute.com/
