# Supplementary material for: A systematic review protocol for assessing equity in clinical practice guidelines for traumatic brain injury and homelessness
Source: Front Med (Lausanne). 2022 Jul 22;9:815660. doi: 10.3389/fmed.2022.815660 (PMC9353519; doi:10.3389/fmed.2022.815660)
Supplement: Supplementary file 4 [file Table_4.pdf]

Chan et al. (2022). A systematic review protocol for assessing equity in clinical practice guidelines for traumatic brain injury and homelessness. *Front. Med.* 9:815660.

Supplementary Material 4 – Study Selection Process for Gray Literature

Table 1. Targeted Websites to Search for Clinical Practice Guidelines

Document the targeted website(s) identified in Grey Matters, by the Research Team, and from Google Search that will be searched for CPGs on TBI or homelessness

#	Date Searched	Name of Website/Organization	Link of Website/Organization	Source *(i.e., Grey Matters, Research Team, Stakeholder, Google Search)*	Search Strategy Applied for Google Search	Number of Items Retrieved/ Identified from Search
1						
2						
3						
4						
5						

Table 2. Stakeholders Contacted

Document the stakeholders who were contacted for targeted websites and/or relevant CPGs

#	Name of Individual/Organization	Date Contacted	Number of Items Recommended	Type of Item *(i.e., Website, CPG)*
1				
2				
3				
4				
5				

Table 3. Clinical Practice Guidelines Identified from Websites

Document the CPGs on TBI or homelessness identified from targeted website(s)

#	Date Searched	Authors of CPG	Title of CPG	Link for CPG	Targeted Website *(i.e., where the CPG was identified)*	Search Strategy Applied to Identify CPG	Number of Items Retrieved/Identified from Search *(i.e., # of CPGs for title and abstract screen)*
1							
2							
3							
4							
5							

Table 4. Title and Abstract Screen of CPGs Identified from Websites

#	Item Number in 'CPGs from Websites' Worksheet *(Note: Used for Tracking Purposes)*	Date Screened	Authors	Title of CPG	Link for CPG	<u>Decision</u> 1= Include/Maybe 0 = Exclude
1						
2						
3						
4						
5						

Table 5. Full-Text Screen of CPGs Identified from Websites

#	Item Number in 'CPGs from Websites' Worksheet *(Note: Used for Tracking Purposes)*	Date Screened	Authors	Title of CPG	Link for CPG	Decision<br>1= Include<br>0 = Exclude	Reason for Exclusion<br>*(e.g., Not homelessness, not TBI, no Full-Text (i.e., summaries/editorials), adaptations/translations, recommendations not rated, recommendations not evidence-based)*
1							
2							
3							
4							
5							
